# Psychological Factors Contributing to Health-Related Quality of Life Following Endometriosis Surgery: Results of a Cross-Sectional Study

**DOI:** 10.1007/s43032-025-01899-3

**Published:** 2025-06-23

**Authors:** Z. Boersen, J. M. Oosterman, C. I. M. Aalders, D. D. M. Braat, C. M. Verhaak, A. W. Nap

**Affiliations:** 1 Department of Gynaecology and Obstetrics, Rijnstate Arnhem, The Netherlands; 2 Donders Institute for Brain, Cognition and Behaviour, Radboud University, Nijmegen, The Netherlands; 3 Department of Obstetrics and Gynaecology, Radboud University Medical Centre, Radboud University, Nijmegen, The Netherlands; 4 Department of Medical Psychology, Radboud University Medical Centre, Nijmegen, The Netherlands

**Keywords:** Catastrophizing, Central sensitization, Chronic pain, Endometriosis, Psychology, Quality of life

## Abstract

**Introduction:**

Endometriosis causes severe and chronic pain leading to impaired Health-Related Quality of Life (HRQoL). While endometriosis surgery does improve pain intensity, psychological factors have an important role in pain perception. The current study aims to evaluate the independent contribution of pain catastrophizing and anxiety and depression to HRQoL between six months and nine years following endometriosis surgery.

**Methods:**

This is a cross-sectional questionnaire study including women with endometriosis who were surgically treated for endometriosis-related pain. Hierarchical multiple linear regression analysis was used to evaluate the relationship of pain catastrophizing and a total score for anxiety and depression to HRQoL, in addition to the contributions of pain intensity, fatigue and sleep quality. In a sub-analysis, we evaluated this relationship in patients with a shorter and with a longer time since surgery (TSS).

**Results:**

The study included 195 participants, revealing significant correlations between HRQoL, pain catastrophizing and anxiety and depression. Subgroup analysis demonstrated that both pain catastrophizing and a combined anxiety and depression score significantly predicted HRQoL in both the shorter and longer TSS groups. These associations were found in addition to the contribution of pain intensity, fatigue and sleep quality to HRQoL.

**Conclusion:**

The current study demonstrates that pain catastrophizing and a combination of anxiety and depression contribute to HRQoL in patients six months to nine years after surgical treatment of endometriosis. Extended post-surgical care could be warranted to address these factors accordingly, for example with psychological care, in addition to surgery alone.

## Introduction

Endometriosis is a condition in which functioning endometrium-like tissue is located outside the uterus. It affects approximately 10% of women in their reproductive age [1]. It can affect multiple tissues and organs such as the gastrointestinal tract, female reproductive organs, or urinary tract [1, 2]. The most common symptom is chronic pain, which impairs Health-Related Quality of Life (HRQoL) [1–3]. To reduce symptoms, treatment usually encompasses pharmacological hormonal suppression. However, when this is not successful, inappropriate, or not wished by the patient, surgery is often necessary, alone or in combination with medication. Depending on the location and the extent of the endometriosis lesions, surgery can be limited to the removal of peritoneal lesions, extraperitoneal endometriosis (e.g. in scar tissue of a caesarian section) and/or removal of ovarian endometriosis cysts. Additionally, in case of deep endometriosis, more extensive surgery may be required, including segmental bowel resection and/or partial bladder resection, often in combination with adhesiolysis. Hysterectomy with or without removal of the adnexa may also be indicated. During surgery the severeness of endometriosis is reported using the revised American Society for Reproductive Medicine (rASRM) score which rates endometriosis from stage I (minimal) to IV (severe). The score is based on the location, depth and extent of the endometriosis lesions. However, the rASRM score does not consistently correlate with the pain intensity reported by patients with endometriosis [4–6]. Therefore, it is essential to consider patients’ symptoms when determining the indication for surgery. Surgery for endometriosis can improve HRQoL significantly for all rASRM stages, but the degree of improvement may vary depending on the stage of the disease, severity of symptoms, type of surgery performed, the surgeons’ experience, success of the surgical eradication of the endometriosis lesions, and comorbidities [7–9]. Moreover, results from follow-up studies have shown that recurrence of pain occurs in up to 40% of women within five years [10–12], even in the absence of visible recurrence of endometriosis lesions [10, 11, 13, 14]. This suggests that other determinants contribute to pain perception and consequently HRQoL, beyond the effects of the physical manifestation of endometriosis itself.

To identify additional determinants of pain perception, it is essential to examine the definitions of pain in more detail, especially chronic pain. Chronic pain is a multidimensional symptom involving alterations in both ascending and descending pain pathways that contribute to pain perception. The ascending pathways are responsible for the processing of peripheral stimuli. They therefore respond, at least initially, to traditional pain mitigation strategies such as analgesics or surgery [15, 16]. Descending pathways involve top-down mechanisms such as genetics, prior experiences, expectations, emotions and mood. Descending pain pathways are important for pain modulation. If these descending pathways become dysregulated due to ongoing pain, the pain processing system can enter an abnormal state of responsiveness [14, 16, 17], defined as central sensitization. This central sensitization to pain leads to a heightened perception of painful and even non-painful stimuli beyond the affected area, instead of reflecting the presence of noxious stimuli [17]. In the case of chronic pain, the fear-avoidance model can be used to further understand and identify factors related to its manifestation and sustainment [18]. This model illustrates how interpreting pain as a threat can initiate a cycle of fear, avoidance, and disability. A key factor in the fear-avoidance model is pain catastrophizing. Pain catastrophizing is a cognitive strategy in which individuals interpret pain as threatening. It encompasses thoughts like “I can’t handle this pain—it’s only going to get worse”. In the fear-avoidance model, pain catastrophizing leads to an exaggerated negative orientation toward pain [3]. Patients who catastrophize tend to avoid activities that could provoke pain, resulting in physical inactivity and isolation. Importantly, this avoidance behavior is reinforced by the relief of emotional distress, which has become the primary driver of continued avoidance, rather than the initial painful stimulus. Compared to healthy controls, endometriosis patients show significantly higher levels of pain catastrophizing [3]. Although results from a prospective study showed that pain catastrophizing improved one-year post-surgery compared to baseline measurements, pain catastrophizing was still associated with higher pain intensity levels in patients with endometriosis [19]. Moreover, pain catastrophizing relates negatively to HRQoL, independent of pain intensity [3]. However, this was not specifically investigated after surgical treatment of endometriosis. Moreover, studies evaluating the relationship between pain catastrophizing and pain only focused on short-term outcomes (one year) or did not report the mean duration post-surgery altogether [19, 20].

Additionally, anxiety and depression [16] can also modulate pain perception, thereby influencing HRQoL. The prevalence of depression and anxiety in women diagnosed with endometriosis has been shown to be higher compared to patients with other chronic pain conditions including chronic low back pain and rheumatoid arthritis [21]. However, the contribution of anxiety and depression to HRQoL has only been evaluated relatively shortly after surgery or has not been investigated at all after surgical treatment of endometriosis [22–26].

Due to the high recurrence rate of pain and the diminished HRQoL after endometriosis surgery, there is a need to identify additional factors contributing to HRQoL, both at short-term and long-term follow-up after surgery. This could provide insight into potential psychological factors that can be targeted to preserve or improve HRQoL in the short- and long-term following surgery.

Therefore, we conducted a cross-sectional study to determine the contribution of pain catastrophizing and anxiety and depression to HRQoL up to nine years following endometriosis surgery. Additionally, a sub-group analysis was performed to explore the contribution of pain catastrophizing and anxiety and depression to HRQoL in patients with shorter and longer post-surgery durations. We hypothesize that pain catastrophizing, and anxiety and depression will independently contribute to HRQoL in patients surgically treated for endometriosis, irrespective of the time that has elapsed since surgery.

## Methods

### Study Population and Recruitment

All women aged 18 years or older at the moment of inclusion who underwent surgery for endometriosis-related pain in the secondary referral center Rijnstate Hospital between 2012 and July 2020 were eligible for this study. The surgical indication in Rijnstate Hospital was established through shared decision-making, taking into account the severity of symptoms, extent of the disease, patient’s current and future reproductive desires and personal preferences. Patients were excluded from the study if no endometriosis was found during surgery or in the removed tissue during histopathological assessment/evaluation post-surgery, or if they had insufficient understanding of the Dutch language making it impossible for them to complete the questionnaires. In January 2021, during the COVID-19 pandemic, all patients meeting the inclusion criteria were contacted by email asking them to participate in this study. The invitation contained a URL which patients could use to log in online and choose whether they wanted to participate in the study. After two weeks, a reminder was sent to all participants who had not responded. After four weeks all patients who had not responded to this reminder were contacted by phone by one of the members of the research team, asking them to participate in the study. Patients for whom no email address was available, received a written invitation and were contacted by phone asking if they were willing to participate in the study. If these patients chose to participate, their email address was collected and they received an electronic invitation as well.

### Measures

#### Demographic and Endometriosis Related Information

A complete overview of the demographic variables that were collected is provided in Table 1. Part of the demographic information was collected from the questionnaires. Additionally, researchers collected the following data from patients’ medical file including age, age at the first endometriosis-related surgery, number of endometriosis-related surgeries, and time that elapsed since the most recent endometriosis-related surgery that was performed in the hospital where the study was conducted (Time Since Surgery or TSS). The Body Mass Index (BMI) of patients was consistently reported in the medical records prior to the most recent endometriosis-related surgery. This enabled us to calculate the BMI change between the most recent surgery and completion of the questionnaires. Furthermore, information about the rASRM classification established during the most recent surgery was collected. In addition, we collected the extent of all endometriosis-related surgeries from patient’ medical records. Because the results from a study showed that the extent of the endometriosis-related surgery could be considered as risk factor for worse surgical outcomes [27], we hypothesized that the extent of the patients’ endometriosis-related surgical history could serve as a potential confounder for HRQoL. To incorporate the extent of the surgery in the analysis, we created a categorization scheme based on the extensiveness of the surgeries. The categorization scheme ranged on a scale from 0 to 5, with a higher score corresponding with more extensive surgery: (0) diagnostic laparoscopy without removal of endometriosis lesions, (1) removal of endometriosis located in the abdominal wall (e.g. located in old cesarean scar tissue or near the umbilical), (2) removal of peritoneal endometriosis and/or adhesiolysis, (3) removal of ovarian endometriosis (cystectomy, tubectomy, ovariectomy), (4) hysterectomy without removal of ovarian endometriosis and (5) hysterectomy with removal of ovarian endometriosis and/or removal of deep endometriosis (e.g. intestinal or bladder endometriosis). If a patient received multiple scores on a single operation (e.g. 2 and 5) or had undergone multiple endometriosis-related surgeries, the highest score was used.

**Table 1:** Patients’ demographics

	Short TSS	Long TSS	Total TSS
N (N (%))	101 (51.8)	94 (48.2)	195 (100.0)
Age years (mean (SD))	39.2 (7.5)	40.5 (7.2)	39.9 (7.4)
Age at first surgery years (mean (SD))	30.4 (6.2)	33.4 (6.8)	31.8 (6.6)
BMI at most recent surgery Kg/m^2^ (mean (SD))	25.5 (4.5)	26.0 (4.8)	25.7 (4.6)
BMI change Kg/m^2^ (mean (SD))	0.0 (2.1)	0.9 (2.4)	0.5 (2.3)
TTS months (mean (SD))	28.0 (12.4)	70.3 (15.1)	48.4 (25.2)
Education level (median (25–75 percentile))	6 (5–6)	6 (5–6)	6 (5–6)
Paid work (N (%))			
* No*	22 (21.8)	15 (16.0)	37 (19.0)
* Yes*	79 (78.2)	79 (84.0)	158 (81.0)
Marital status (N (%))			
*Single*	13 (12.9)	9 (8.0)	23 (11.8)
* Married/living with partner*	83 (82.2)	97 (86.6)	160 (82.1)
* Separated*/*divorced*	4 (4.0)	5 (4.5)	11 (5.6)
* Widow*	1 (1.0)	1 (0.9)	1 (0.5)
Nullipara (N (%))			
* No*	57 (56.4)	55 (58.5)	112 (57.4)
* Yes*	44 (43.6)	39 (41.5)	83 (42.6)
Current use of analgesics (N (%))			
*No*	56 (55.4)	60 (63.8)	123 (63.1)
* Yes*	38 (37.6)	34 (36.2)	72 (36.9)
Current use of hormonal medication (N (%))			
* No*	56 (55.4)	55 (58.5)	111 (56.9)
* Yes*	45 (44.6)	39 (41.5)	84 (43.1)
Extensiveness of surgery (N (%))			
* Removal of extra peritoneal endometriosis*	1 (1.0)	1 (1.1)	2 (1.0)
* Removal of peritoneal endometrioses and/or adhesiolysis*	8 (7.9)	6 (6.4)	14 (7.2)
* Removal of ovarian endometriosis*	11 (10.9)	13 (13.8)	24 (12.3)
* Hysterectomy without removal of ovarian endometriosis*	25 (24.8)	24 (25.5)	49 (25.1)
* Hysterectomy with removal of ovarian endometriosis and/or removal of deep endometriosis*	3 (3.0)	6 (6.4)	9 (4.6)
rASRM score (median (25–75 percentile))	44 (46.8)	97 (49.7)	3 (2–4)
Number of endometriosis related surgeries (median (25–75 percentile))	2 (1–3)	2 (1–3)	2 (1–3)

*TSS *Time Since surgery; *BMI *Body Mass Index; *rASRM *revised American Society for Reproductive Medicine. Patients’ educational attainment was scored using a 7 point rating scale [39]: (1) unfinished primary school, (2) finished primary school, (3) unfinished low-level secondary education, (4) lower vocational training, (5) advanced vocational training or lower professional education, (6) finished higher professional education or senior general secondary education, and (7) obtained a university degree

#### HRQoL

Participants were asked to complete two questionnaires evaluating HRQoL: the Endometriosis Health profile (EHP-30) and the Dutch version of the Short Form 36 (RAND-36). The EHP-30 is a disease-specific HRQoL questionnaire which has been validated for use in Dutch endometriosis patients [28, 29]. It measures the impact of the disease on physical, mental, and social aspects of daily life. The questionnaire is divided into two parts. The core questionnaire consists of five subscales: pain, control and powerlessness, emotional well-being, social support, and self-image. The second part consists of six subscales: work, relationship with children, sexual intercourse, infertility, medical profession, and treatment. The EHP-30 ranges from 0 to 100 with a higher score corresponding to worse HRQoL. In the analysis we used both parts of the EHP-30. To measure general HRQoL, we used the validated standardized RAND-36 [30] version 2.0. The RAND-36 is a multipurpose, general health survey which is applied to measure HRQoL in nine different domains: physical functioning, social functioning, role limitations due to physical health, role limitations due to emotional problems, emotional well-being, vitality, pain, general health, and health change. The RAND-36 score ranges from 0 to 100 with a higher score corresponding to a better HRQoL.

#### Pain Catastrophizing

Pain catastrophizing was measured using the validated Pain Catastrophizing Scale (PCS) [31]. The PCS evaluates pain catastrophizing by measuring feelings of rumination, magnification, and helplessness, and consists of 13 items reflecting on patients’ thoughts and feelings during a painful experience on a 5-point Likert scale. Scores can range from 0 to 52 with a higher score corresponding to more catastrophizing. A score higher than 30 is usually considered as clinically relevant catastrophizing.

#### Anxiety and Depression

Anxiety and depression symptoms were measured by the Hospital Anxiety and Depression Scale (HADS). The HADS has been validated for a variety of conditions such as lupus erythematosus and chronic fatigue syndrome [32, 33] and contains 14 questions which patients can score on a 4-point Likert scale. In this questionnaire, a set of seven questions rate feelings of anxiety and seven questions rate feelings of depression. The HADS depression and anxiety scores can range from 0 to 21 with a higher score indicating worse symptoms. A score from 0 to 7 on corresponds with no anxiety or depression. Scores of 8 to 10 and 11 to 21 correspond with possible and likely anxiety or depression, respectively.

#### Pain Intensity

To measure pain intensity, patients were asked to complete a Numerical Rating Scale (NRS). This scale is widely used in a variety of chronic pain conditions such as low back and neck pain [34, 35]. Specifically, patients were asked to perform two ratings, one evaluating the overall worst and one evaluating the overall average pain that patients had experienced over the past seven days. Both items were scored on a scale ranging from 0 (no pain) to 10 (worst pain possible).

#### Sleep Quality and Fatigue

Sleep quality and fatigue were measured with an NRS over the past four weeks. Sleep quality [36] ranged from 0 (very poor sleep quality) to 10 (excellent sleep quality). Fatigue [37] was also measured ranging from 0 (no fatigue) to 10 (worst fatigue imaginable).

### Data Analysis

In order to identify the independent contribution of the PCS and HADS scores to the HRQoL score, the following steps were taken.

First, an average score for HRQoL was calculated. For this, the subscales of the EHP-30 and RAND-36 were separately averaged into an EHP-30 and RAND-36 total score. Then both the RAND-36 total score and the EHP-30 total score were standardized by calculating z-scores. For several participants, no scores on the EHP-30 part one were available (*n* = 38); in those instances, only the available data (e.g., EHP-30 part 1 and/or 2 and/or RAND-36 scores) were used. Next, the EHP-30 score was multiplied by −1 so that a higher score indicated better HRQoL, after which the RAND-36 and EHP-30 questionnaires were averaged into a single HRQoL score. This score was used in the analysis as an indicator for HRQoL. This way, we combined the general HRQoL score as measured by the RAND-36 together with the disease specific HRQoL measured by the EHP-30, to get a more comprehensive assessment of HRQoL. At the same time this also reduced the number of separate analyses needed, a decision supported by the good reliability of the combined HRQoL domain score (Cronbach’s alpha 0.0816).

Next, we assessed which factors contributed to HRQoL. For this, we performed hierarchical multiple linear regression analyses with bootstrapping (1000 samples). In the first step, potential confounding variables were entered (Table 2). In the second step, the NRS scores for pain intensity, fatigue, and sleep quality were entered. In the final step, PCS and HADS scores were entered. To avoid multicollinearity, we calculated an average NRS score for pain intensity by adding the mean and worst pain intensity scores and dividing them by two. For the same reason we used the total HADS score instead of the two individual subscales for anxiety and depression.

**Table 2:** Spearman’s rank correlations between potential confounders and HRQoL

	Correlation coefficient		
	Short TSS	Long TSS	Total TSS
Age	.335*	.360*	.358*
Age at first surgery	-.093	-.038	-.040
BMI change	-.048	-.178	-.111
TSS	-	-	.107
Education level	.047	-.017	.017
Paid work	.276*	.355*	.312*
Nullipara	-.069	-.189	-.127
Current use of analgesics	-.490*	-.474*	-.486*
Current use of hormonal medication	-.238*	-.105	-.180*
Extensiveness of surgery	.224*	.033	.131
Number of endometriosis related surgeries	.125	.075	.098

^*^ = Significant; *HRQoL* Health-Related Quality of Life; *TSS* Time Since Surgery; *BMI* Body Mass Index

In a sub-analysis, we used hierarchical multiple linear regression to evaluate if the PCS and HADS total scores contributed to HRQoL both in patients with a shorter and longer post-surgery period. For this, we divided the study sample into two equal groups based on the median TSS: 6 to 47 months (group with a shorter time since surgery; STSS) and 48 to 108 months (group with a longer time since surgery; LTSS) so that both groups have a comparable sample size. This enabled us to investigate the relationship and direction between HRQoL and the predictors of interest for different post-operative TSSs.

For the potential confounding variables, we selected factors based on the literature and expert opinion of the researchers, namely age, age at first surgery, change in BMI over post-surgery duration, paid work, current use of analgesics and current use of hormonal medication, nulliparity and extensiveness of the endometrioses-related surgeries [38]. Education, measured with an ordinal scale, was divided into ‘low’ (score 1–4, reflecting less than primary education to lower secondary education), ‘middle’ (score 5, indicating secondary vocational education) and ‘high’ (score 6 & 7, reflecting higher secondary and higher vocational education, and university degree) education [39]. Because most participants in this study reported middle or high education, leaving a limited number of patients with score 1 to 4 (*n* = 16), we combined the low and middle scores (scores 1–5) into a single ‘low’ variable. The variable “high education” still consisted of scores 6 and 7. Next, a binary variable was created with low education scored as 0 and high education scored as 1. The analysis did not include the rASRM-score as a potential confounder because it primarily assesses anatomical findings and has been shown to poorly correlate with patient-reported symptoms [5]. To reduce the number of confounding variables entered in the regression model, we first explored which potential confounding demographic and clinical factors contributed to HRQoL in a separate Spearman’s rank correlation analysis. The TSS was only included in the explorative confounder analysis of the first, main analysis, because in the subgroup analysis we split the group in two based on the TSS. Factors that significantly correlated with HRQoL were considered to be confounders and were therefore included in the first step of the regression analyses.

All analyses were conducted using the SPSS software package (version 29). The statistical significant level was set at p ≤ 0.05.

## Results

**Fig. 1:**
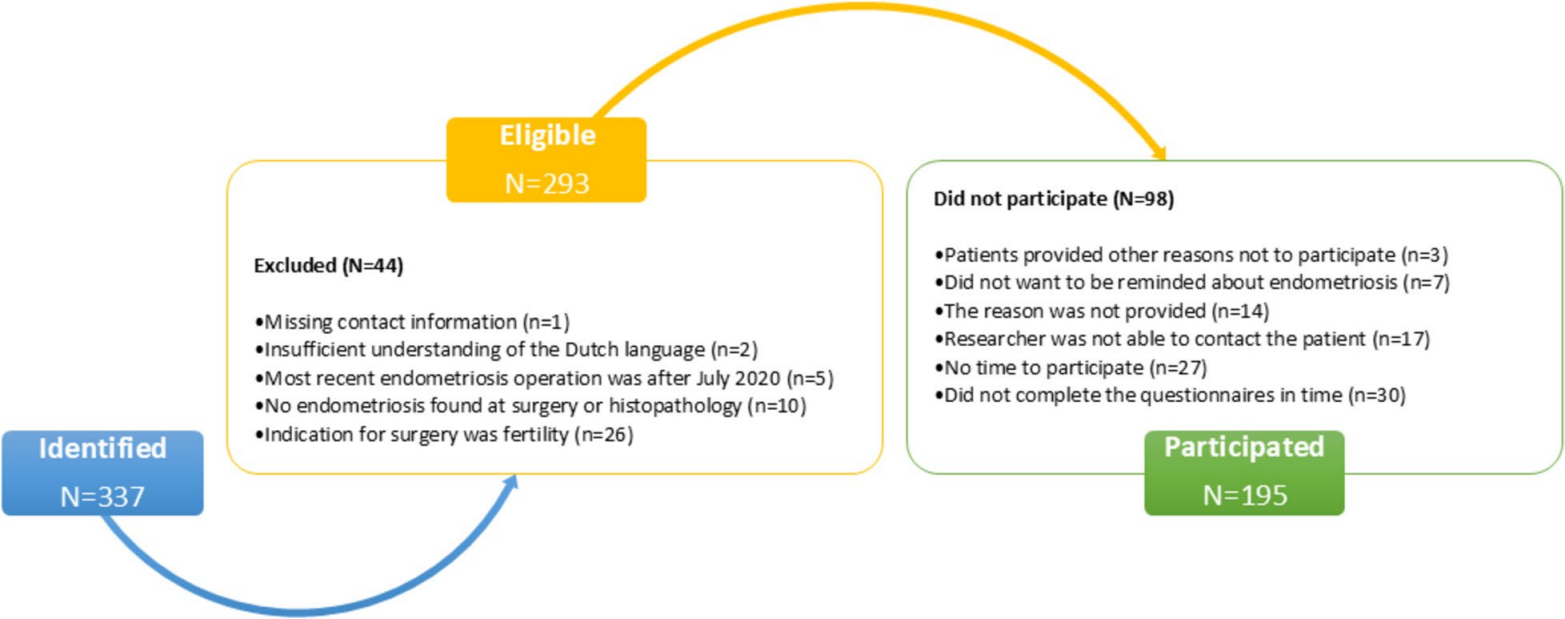
Patient flow throughout the study

A total number of 337 patients who underwent endometriosis surgery between January 2012 and July 2020 were identified. Of these, 293 patients were eligible for inclusion. Reasons for exclusion were: indication for surgery was only fertility related (*n* = 26), missing contact information (*n* = 1), the most recent endometriosis operation was after July 2020 (*n* = 5), no endometriosis was found during surgery or in histopathological investigation post-surgery (*n* = 10), or patients were unable to complete the questionnaires due to insufficient understanding of the Dutch language (*n* = 2). Two thirds (*n* = 195; 66.6%) of eligible patients participated in this study. Reasons for the 98 patients who did not participate were diverse. Some patients did not complete the questionnaires in time (*n* = 30). Others reported that they had no time to participate (*n* = 27) or did not want to be reminded about endometriosis (*n* = 7). In some cases the researcher was unable to contact the patient (*n* = 17), or the reason was not provided (*n* = 14). Three patients provided other reasons to refrain from participation in this study. Figure 1 contains an overview of patient flow throughout the study.

Demographic data show that the mean age of the participants was 39.9 (SD 7.4) years, while the mean age at first surgery of the participants was 31.8 (SD 6.6) years. The median education level according to Verhage [39] was 6, corresponding with finished higher professional education or senior general secondary education. Most women were married or living with a partner (82.1%). Average BMI prior to the most recent surgery was 25.7 (SD 4.6), whereas since then the BMI increased 0.5 (SD 2.3) on average. In addition, 42.6 percent of women were nullipara. Almost two thirds of patients (63.1%) reported that they did not currently use any analgesics medication and 56.9% did not currently use any hormonal medication. In 97 patients (49.7%) the surgery consisted of removal of deep endometriosis and/or hysterectomy with removal of ovarian endometriosis. The median number of surgeries was 2. The median rASRM score was 3, corresponding with moderate endometriosis. Mean TSS was 48.4 (SD 25.2) months. A complete overview of the demographic data is provided in Table 1 and mean scores of the questionnaires are presented in Table 3.

**Table 3:** Patients’ mean scores to questionnaires regarding Health-Related Quality of Life, stress, anxiety, depression, pain and fatigue

Mean (SD)					
			STSS	LTSS	Total
RAND-36^a^					
* Physical*^b^			69,8 (23,2)	73,3 (23,0)	71,5 (23,1)
* Mental*^*b*^			69,3 (22,5)	71,7 (21,9)	70,5 (22,2)
EHP- 30^a^					
* Core*^c^			30.1 (22.2)	28.9 (21.3)	29.6 (21.7)
* General*			27.0 (20.9)	22.6 (19.6)	24.9 (20.4)
PCS			13.8 (12.2)	11.2 (12.1)	12.6 (12.2)
HADS					
* Anxiety*			5.7 (4.6)	4.9 (4.3)	5.3 (4.4)
* Depression*			4.2 (4.4)	3.9 (4.0)	4.1 (4.2)
* Combined*			9.9 (8.6)	8.8 (7.6)	9.3 (8.1)
NRS					
* Average*			3.0 (2.6)	2.4 (2.5)	2.7 (2.6)
* Worse*			3.6 (3.2)	3.2 (3.1)	3.4 (3.1)
* Combined*			3.3 (2.9)	2.8 (2.8)	3.0 (2.8)
Fatigue			5.1 (2.4)	4.9 (2.7)	5.0 (2.6)
Sleep quality			6.0 (2.1)	6.0 (2.1)	6.0 (2.1)

*STSS* shorter Time Since Surgery; *LTSS* longer Time Since Surgery; *EHP* Endometriosis Health Profile; *RAND* Research And Development; *NRS* Numerical Rating Scale; *PCS* Pain Catastrophizing Scale; *HADS* Hospital Anxiety and Depression Scale; *SD* Standard deviation; ^a^A higher score of the RAND-36 corresponds with better Health-Related Quality of Life, and a lower score of the EHP-30 corresponds with better Health-Related Quality of Life; ^b^Missing data *n* = 2; ^c^Missing data *n* = 38

### Hierarchical Multiple Regression Analysis

The assumptions for linear regression regarding linearity, homoscedasticity and independence were all met; no severe violations of normality were observed, and Bootstrapped results are reported. Furthermore, the Variance Inflation Factor (VIF) was lower than 3 between variables indicating low multicollinearity (a VIF score above 5 is considered to indicate severe multicollinearity).

To assess which confounders significantly related to HRQoL, we first performed a correlation analysis. The results are presented in Table 3. This showed that age, paid work, the current use of pain and hormonal medication all significantly correlated with HRQoL. These variables were therefore included as confounders in the first step of the multiple regression analysis. The second step showed that, in addition to the confounders, the NRS scores for pain intensity (B = −0.162; *p* < 0.001) and fatigue (B = −0.084; *p* < 0.001) significantly correlated with HRQoL. However, sleep quality did not. The PCS (B = −0.016; *p* < 0.001) and HADS total (B = −0.039; *p* < 0.001) scores, entered in the last step, both significantly contribute to HRQoL and higher scores on these factors were related to a lower HRQoL score. Together these two variables explained 10% of the variance in HRQoL. An overview of the analysis is presented in Table 4.

**Table 4:** Multiple hierarchical regression analyses

		B	Significance (2-tailed)	95% Confidence Interval		ΔR^2^	F
				Lower	Upper		
Total TSS^1^							
Model 2	*Pain intensity*	-.162	<.001*	-.196	-.128		
	*Fatigue*	-.084	<.001*	-.129	-.046		
	*Sleep quality*	.038	.078	-.005	.078		
						.321	65.648*
Model 3	*Pain intensity*	-.111	<.001*	-.144	-.078		
	*Fatigue*	-.031	.060	-.063	.002		
	*Sleep quality*	.003	.864	-.032	.038		
	*Pain catastrophizing*	-.016	<.001*	-.024	-.009		
	*Anxiety and depression*	-.039	<.001*	-.049	-.029		
						.104	89.497*
Short TSS^2^							
Model 2	*Pain intensity*	-.191	<.001*	-.232	-.146		
	*Fatigue*	-.089	.006*	-.143	-.028		
	*Sleep quality*	.034	.219	-.019	.090		
						.351	35.014*
Model 3	*Pain intensity*	-.142	<.001*	-.175	-.101		
	*Fatigue*	-.029	.284	-.076	.027		
	*Sleep quality*	.004	.844	-.041	.043		
	*Pain catastrophizing*	-.011	.041*	-.022	-.001		
	*Anxiety and depression*	-.042	<.001*	-.056	-.026		
						.089	46.502*
Long TSS^3^							
Model 2	*Pain intensity*	-.128	<.001*	-.188	-.078		
	*Fatigue*	-.083	.005*	-.130	-.029		
	*Sleep quality*	.043	.186	-.023	.098		
						.278	29.386*
Model 3	*Pain intensity*	-.080	.006*	-.141	-.022		
	*Fatigue*	-.032	.133	-.068	.016		
	*Sleep quality*	.017	.565	-.035	.082		
	*Pain catastrophizing*	-.024	<.001*	-.036	-.013		
	*Anxiety and depression*	-.034	<.001*	-.054	-.018		
						.126	40.556*

^*^ statistically significant (p ≤ 0,05); *TSS* Time Since Surgery; *BMI* Body Mass Index; Confounders included in the analysis were: (1) Age, Paid work, Use of analgesics, Use of hormonal medication; (2) Age, Paid work, Use of analgesics, Use of hormonal medication Extensiveness of surgery; (3) Age, Paid work, Use of analgesics

### Sub-Group Analysis

For both sub-groups we used the correlation analysis to determine which confounders needed to be included in the regression analyses (Table 3). In the STSS group, the NRS score for pain intensity (B = −0.191; *p* < 0.001) and fatigue (B = −0.089; p < 0.001) significantly correlated with HRQoL. In addition, PCS (B = −0.011; *p* = 0.041) and HADS total (B = −0.042; *p* < 0.001) scores showed a significant, negative association with HRQoL. These two variables explained 9% of the variance of HRQoL, in addition to the variables entered in the first and second step. The analysis for the LTSS group showed that the NRS scores for pain intensity (B = −0.128; *p* < 0.001) and fatigue (B = −0.083; *p* = 0.005) significantly contributed to HRQoL. In addition, the scores for PCS (B = −0.024; *p* < 0.001) and HADS (B = −0.034; *p* < 0.001) also significantly contributed to HRQoL. The extra variance explained by these two variables of interest was 13%. The NRS score for sleep quality did not significantly contribute to HRQoL in either the STSS or LTSS group. Table 4 contains an overview of the analyses.

## Discussion

The goal of the present study was to evaluate the independent contribution of pain catastrophizing together with anxiety and depression to HRQoL in patients who underwent their most recent endometriosis-related surgery within the past nine years. In particular, the contribution of anxiety and depression to the HRQoL was analyzed in the same comparison subcategory. The results of all three analyses showed that pain catastrophizing, and the combined anxiety and depression score negatively contributed to HRQoL independent of pain intensity, fatigue, and sleep quality.

While pain catastrophizing and the combined score of anxiety and depression contributed to HRQoL independent of pain intensity, pain intensity did contribute to HRQoL in all three analyses. Notably, post-surgical pain scores in our sample were low—3.3 in the STSS group and 2.8 in the LTSS group—below the commonly used clinical threshold for initiating medical treatment [40]. These results demonstrate that chronic pain is a multifaceted symptom, challenging to treat, and contributes to low HRQoL even after surgical treatment of endometriosis. Based on our results, pain catastrophizing is an interesting factor because it independently contributes to HRQoL at total, short and long TSS. The contribution to HRQoL could be explained within the context of the fear-avoidance model [19, 41]. Based on this model, it can be speculated that patients with endometriosis continue to catastrophize after surgical treatment, which results in avoidance of activities that might evoke pain, and isolation. Ultimately, this contributes to a reduced HRQoL. We speculate that addressing pain catastrophizing with a targeted treatment has the potential to further improve HRQoL after the surgical treatment of endometriosis-related chronic pain.

In correspondence with other studies, the results from this study show that higher levels of anxiety and depression correlated with lower HRQoL [42]. Additionally, our results reveal that these associations were true for all TSSs and that higher levels of anxiety and depression contributed to HRQoL directly, independent of pain intensity, fatigue and sleep quality. However, anxiety and depression might also indirectly influence HRQoL by influencing the perception of pain. The perception of pain can be modulated by a variety of factors like prior experiences, expectations, emotions and mood [14, 16, 17]. Therefore, higher levels of anxiety and depression could reduce the down-regulation of the pain processing system, increase perception of pain intensity and reduce HRQoL. Speculatively, reducing anxiety and depression could both directly and indirectly (via its effect on pain) influence HRQoL.

Both pain catastrophizing and anxiety and depression can be targeted with psychological therapy. In chronic pain conditions such as back pain and irritable bowel syndrome, psychological interventions proved to be effective in reducing pain and improving HRQoL [43–45]. To date, there is emerging evidence that psychological interventions could help endometriosis patients as well [46]. Therefore, a psychological intervention, aimed at improving catastrophizing, pain cognitions and mood, can be considered in all women with endometriosis-related chronic pain symptoms. Even when a patient does not show any clinical signs of an anxiety or depression disorder, the results from this study show that these factors can still influence HRQoL. This psychological therapy should be combined with either pharmacologically induced suppression or surgical removal of the endometriotic lesions [47].

### Strengths and Limitations

An important strength of this study is the long post-surgery duration of up to nine years, not often reported in literature. This duration enabled us to evaluate the relation between HRQoL and its predictors up to nine years after surgical treatment of endometriosis. This long duration strengthens the conclusions of this study about the importance of psychological factors in the management of HRQoL in endometriosis patients over time. Another strength is the fact that all patients were selected by evaluating their medical records, providing detailed and reliable information from the participants’ medical records. The group of participants is potentially smaller than when selection was conducted by online recruitment via patient support groups, but it enabled us to verify the medical data using patients’ medical records. If patients are asked to report medical data themselves, this introduces bias even risking to include patients that were not diagnosed with endometriosis at all.

This study has some limitations too. The most important limitation is that we do not have data about pre-surgical and early post-surgical HRQoL and factors that may influence this HRQoL. This would have provided important additional information and insights including the effect of surgery on these factors, how these variables have changed over time and if the relationship and/or direction between HRQoL and the predictors changed. Regardless, our results are valuable and hold promise to catalyze further research initiatives. Another limitation is that we averaged the NRS scores and combined the HADS subscales, which limits the ability to distinguish the individual effects of mean pain, worst pain, anxiety, and depression. However, this was necessary to avoid multicollinearity in the analysis. In addition, the surgeons’ experience is a major determinant in the postoperative surgical outcome and recovery. Unfortunately, we had limited information regarding the surgeon’s experience dating back to 2012. Therefore, this was not included in the analysis. Finally, HRQoL and all the other questionnaires were completed by patients during the COVID-19 pandemic, at a time when COVID-restrictions were harsh in the Netherlands: people were forced to stay at and work from home, schools were closed as well as restaurants, stores, and museums. Although not evaluated in this study, it could be hypothesized that this influenced the HRQoL and/or the pain and psychological complaints reported by patients.

## Conclusions

Taken together, the current study demonstrates that pain catastrophizing and anxiety and depression show an independent negative contribution to the HRQoL in patients six months to nine years after surgical treatment of endometriosis. More research is warranted to assess the interplay between these factors, the effect of surgery and HRQoL, and the potential benefits of an integrated psychological and surgical interventions aimed at improving HRQoL.

## Data Availability

Original data will be available on request (10.17026/dans-x8v-gnhy) in accordance with the conditions of ethics approval. If participants wish, they will be notified of the findings when they are available.
